# Emerging Gene and Small Molecule Therapies for the Neurodevelopmental Disorder Angelman Syndrome

**DOI:** 10.1007/s13311-021-01082-x

**Published:** 2021-09-15

**Authors:** Nycole A. Copping, Stephanie M. McTighe, Kyle D. Fink, Jill L. Silverman

**Affiliations:** 1 School of Medicine, Department of Psychiatry and Behavioral Sciences, MIND Institute, University of California, Research II Building 96, 4625 2nd Avenue, Suite 1001B, Davis, Sacramento, CA 95817 USA; 2 Arkuda Therapeutics, 200 Arsenal Yards Blvd, Suite 220 , Watertown, MA USA; 3 Stem Cell Program and Gene Therapy Center, Department of Neurology, MIND Institute, University of California, Davis, Sacramento, CA USA

**Keywords:** Angelman syndrome, Seizures, Gene therapy, Animal models, Antisense oligonucleotides, Stem cells, Precision medicine, Delivery, Preclinical, Small molecules, Pharmacology, Treatment

## Abstract

Angelman syndrome (AS) is a rare (~1:15,000) neurodevelopmental disorder characterized by severe developmental delay and intellectual disability, impaired communication skills, and a high prevalence of seizures, sleep disturbances, ataxia, motor deficits, and microcephaly. AS is caused by loss-of-function of the maternally inherited *UBE3A* gene. *UBE3A* is located on chromosome 15q11–13 and is biallelically expressed throughout the body but only maternally expressed in the brain due to an RNA antisense transcript that silences the paternal copy. There is currently no cure for AS, but advancements in small molecule drugs and gene therapies offer a promising approach for the treatment of the disorder. Here, we review AS and how loss-of-function of the maternal *UBE3A* contributes to the disorder. We also discuss the strengths and limitations of current animal models of AS. Furthermore, we examine potential small molecule drug and gene therapies for the treatment of AS and associated challenges faced by the therapeutic design. Finally, gene therapy offers the opportunity for precision medicine in AS and advancements in the treatment of this disorder can serve as a foundation for other single-gene neurodevelopmental disorders.

**Supplementary Information:**

The online version contains supplementary material available at 10.1007/s13311-021-01082-x.

## Introduction

Angelman syndrome (AS) is a rare (~1:15,000) neurodevelopmental disorder characterized by severe developmental delay and intellectual disability, impaired communication skills, and a high prevalence of seizures, sleep disturbances, ataxia, and motor deficits [1, 2]. AS is generally diagnosed in patients over the age of one, as its behavioral characteristics become more readily pronounced and distinct compared to other developmental disorders [3]. Seizures are highly prevalent in AS and occur in over 80% of the population [4]. Seizures typically start early in life and are often (~ 1/3) resistant to classic antiepileptic drugs. They continue throughout an individual’s lifetime and present across multiple seizure types including, but not limited to, absence, myoclonic, and generalized clonic-tonic seizures [5, 6]. Given their frequency and treatment resistance, seizures in AS contribute to significantly higher burden of care [7]. Currently, there is no cure for AS and the only treatments available are those designed to temporarily mitigate symptoms throughout a patient’s lifetime.

AS is caused by loss-of-function of the maternally inherited *UBE3A* gene [8–11]. *UBE3A* is located on chromosome 15q11–13 and is biallelically expressed throughout the body but only maternally expressed in the brain due to imprinting [12–14]. The paternal copy is silenced by a long (> 600 kb) non-coding RNA antisense transcript referred to as the *UBE3A*-ATS [15–17]. Several genetic etiologies lead to AS including de novo interstitial deletions of the maternal allele (~ 65–70% of the AS population), loss-of-function mutations in the maternal allele (5–11%), uniparental disomy resulting in two normal functioning paternal alleles (3–7%), and various imprinting defects (3%) [18–20].

Single-gene disorders are good candidates for precision gene and cellular therapies, and AS is particularly encouraging given the unique properties of *UBE3A*. The presence of a silent, yet functional copy of *UBE3A*, on the paternal allele, allows for the development of therapies directed at reactivation of the paternal copy of *UBE3A* [21, 22]. Moreover, due to the neuronal-specific monoallelic expression of *UBE3A*, therapies aimed at introducing a functional copy of the gene are only required the central nervous system [12, 14]. *UBE3A* is highly conserved across species, making it possible to not only model the disorder but to test various preclinical therapies for safety, efficacy, and toxicity [23–27]. With recent advancements made in the field of gene and cellular therapies, as well as their delivery mechanisms, a potential cure for AS is promising [28–31]. In equally rigorous pursuit are strategies aimed to either correct the cellular pathways and downstream targets disrupted by the loss of *UBE3A*, which are theorized to be more likely symptomatic treatments, in comparison to comprehensive disease modifying therapies. Here, we review *UBE3A* and its contribution to AS, evaluate the strengths and weaknesses of current translational rodent models and how they will help in the transition from preclinical to clinical trials, the applicability and feasibility of emerging gene and cellular therapies for AS, as well as small molecule pharmacological approaches, and challenges that face the field of precision medicine as therapies are developed.

## UBE3A Function 

*UBE3A* encodes for a 100 kDa ubiquitin-protein ligase E3A and was initially described as an association factor between p53 and the E6 oncoprotein of various human papillomavirus types where the E3 ubiquitin ligase binds to p53 and breaks it down using the ubiquitin proteolysis system [32]. The gene spans approximately 120 kb and encodes multiple isoforms that may allow for varying substrate specificity, multiple functions and unique cellular localization patterns [33, 34]. UBE3A is a member of the HECT (homologous to E6-AP COOH-terminus) family of enzymes and acts to transfer activated ubiquitin to a protein, signaling it for degradation by the proteolysis system [35]. Interestingly, many of the missense and single amino acid insertion or deletion mutations seen in AS affect the C-terminal catalytic domainf [36, 37]. UBE3A also plays a role as a non-specific transcriptional coactivator of nuclear hormone receptors, independent of its ligase activity, as mutations affecting E6-AP activity do not change coactivation ability [38, 39].

While the function of UBE3A as a ubiquitin ligase protein and transcriptional coactivator is evident, the exact pathogenesis resulting from loss-of-function of the maternal allele in AS remains elusive. Interestingly, many of the missense and single amino acid insertion or deletion mutations seen in AS individuals affect the C-terminal catalytic domain, thus inferring primary ligase function, as central to mechanism [36, 37]. There is a strong correlation between loss of the E6-AP ligase activity and AS [40] as well as numerous protein targets involved in cell proliferation and survival, synaptic function, cell signaling, and nervous system development that have been identified as UBE3A substrates [41–43]. UBE3A even acts as its own substrate [44]. Moreover, *UBE3A* expression is correlated with the regulation of various genes involved in protein catabolism, cell cycle, brain morphology, and transcriptional regulation [45, 46]. Contributing to its complexity UBE3A is localized in pre- and post-synaptic neurons and localized to either cytoplasm or nucleus by isoform permitting for the widespread functional nature of the protein [47].

## Imprinting of 15q11–13

**Fig. 1:**
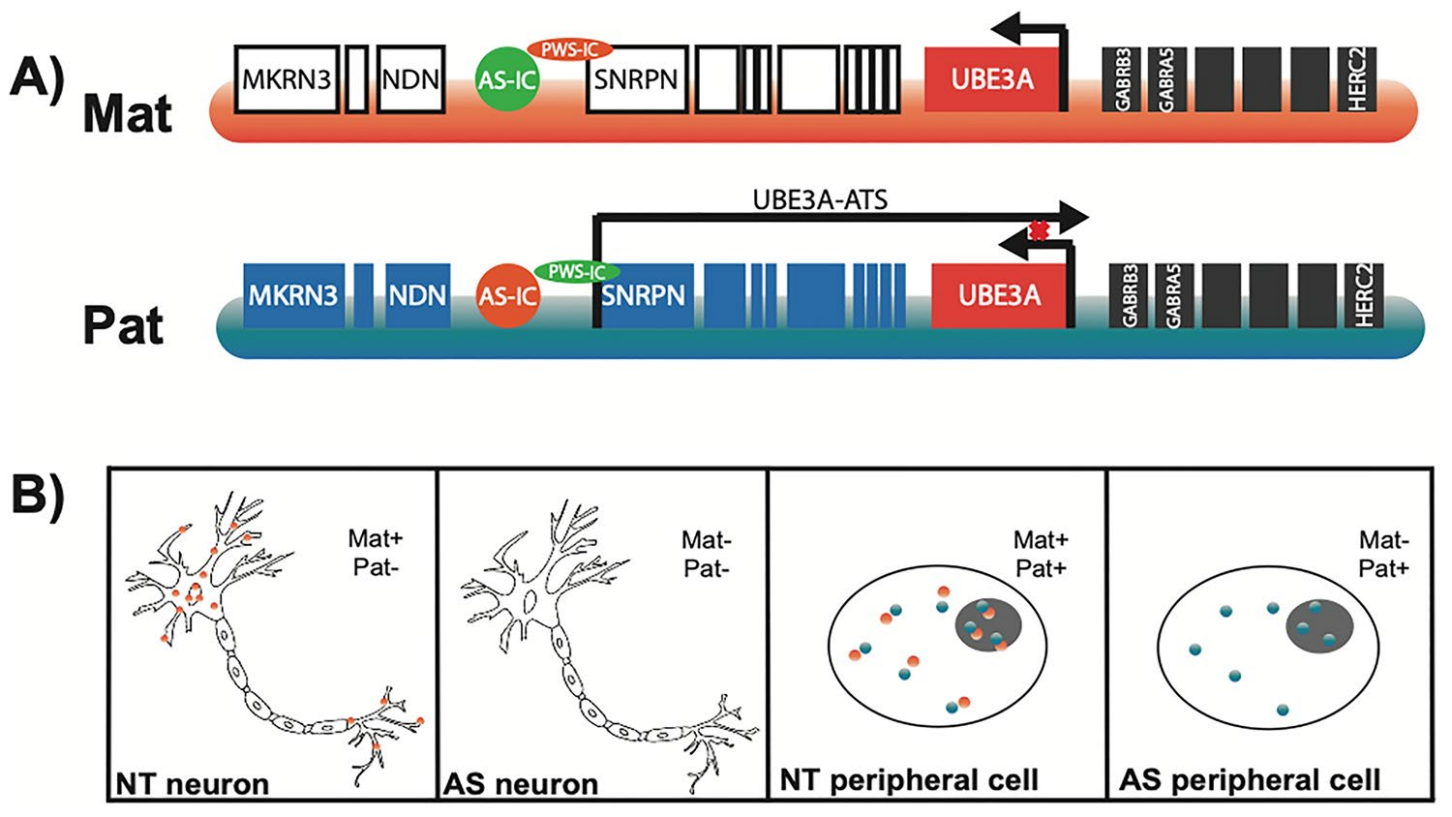
Chromosome 15q11–13 and neuronal epigenetic silencing of *UBE3A*. **A** Schematic of the genomic region of 15q11–13. Blue-shaded genes are paternally expressed, red-shaded genes are maternally expressed, and grey-shaded genes are biallelically expressed. White-shaded genes are silenced on the maternal copy. The PWS-IC is methylated on the maternal allele (orange) and unmethylated on the paternal allele (green) while the AS-IC on both alleles is unmethylated. **B** Cartoon of maternal/paternal UBE3A expression in neurons and somatic cells of neurotypical (NT) individuals and those with Angelman syndrome (AS)

Imprinting is an important epigenetic regulatory mechanism that results in the differential expression of maternal and paternal alleles in a parent-of-origin-dependent manner and is a critical factor in AS. This process occurs in the germline where methyl groups are attached to DNA in segments that are rich in cytosine-guanine dinucleotides, creating an imprinting region that regulates expression [48]. The q11–13 region of chromosome 15 spans a cluster of imprinted genes, including *UBE3A*, that are either maternally or paternally expressed, and have been linked to multiple neurodevelopmental disorders [49]. Within q11–13, there are two critical imprinting regions known as the Prader–Willi syndrome imprinting center (PWS-IC) and the Angelman syndrome imprinting center (AS-IC). The PWS-IC is methylated on the maternal allele and represses expression of genes upstream of *UBE3A* including *MKRN3* (makorin/ring finger protein 3), *NDN* (Necdin), *MAGEL2* (melanoma antigen gene family member like 2), and *SNRPN* (small ribonucleoprotein polypeptide N), while the PWS-IC is unmethylated on the paternal allele allowing for expression of those same genes. The AS-IC is located upstream of the PWS-IC and has been thought to help mediate the switch from paternal to maternal imprinting during oogenesis and act as a bipartite regulatory element with the PWS-IC [50]. Genes downstream of *UBE3A* including *GABRB3*, *GABRA5*, and *HERC2* are not imprinted and biallelically expressed on both the maternal and paternal allele (Fig. 1A). Deletion, uniparental disomy, UBE3A missense coding errors, and imprinting etiologies of AS are characterized, in part, by the loss of methylation at SNRPN, suggesting the importance of epigenetic imprinting during development.

UBE3A is biallelically expressed throughout the body, but only maternally expressed in the central nervous system, due to silencing of the paternal allele by the *UBE3A*-ATS. Therefore, individuals with AS who lack a functional copy of maternal *UBE3A* have no UBE3A expression in the brain while maintaining expression of UBE3A in their periphery (Fig. 1B). The *UBE3A*-ATS is a non-coding, polycistronic transcription unit that initiates at the PWS-IC and/or the *SNRPN* promotor and terminates at the *UBE3A* promotor or ~40 kb beyond the promotor [17, 51, 52]. The exact method of how paternal *UBE3A* is silenced through *UBE3A*-ATS is not fully understood; however, several mechanisms have been suggested including a transcriptional interference mechanism where RNA polymerases of the *UBE3A*-ATS and the gene conflict and disrupt transcription and a RNA interference mechanism where double-stranded RNA forms between the sense and antisense RNAs [53, 54]. Studies have shown that expression of *UBE3A*-ATS is sufficient to silence paternal *UBE3A* and reduction of the *UBE3A*-ATS results in normal paternal expression [55]. Several attempts have been made to suppress the antisense transcript, including artificial transcription factors (ATFs) [56, 57], antisense oligonucleotides (ASOs) [58] and pro-methylation dietary supplements such as folic acid and betaine [59, 60], with some successes adding to evidence that precise molecular therapies are necessary to reactivate the paternal allele in AS. In fact, two ASO compounds are in phase I clinical trials (GeneTx NCT04259281; Roche NCT04428281).

## Preclinical Models of Angelman Syndrome

**Fig. 2:**
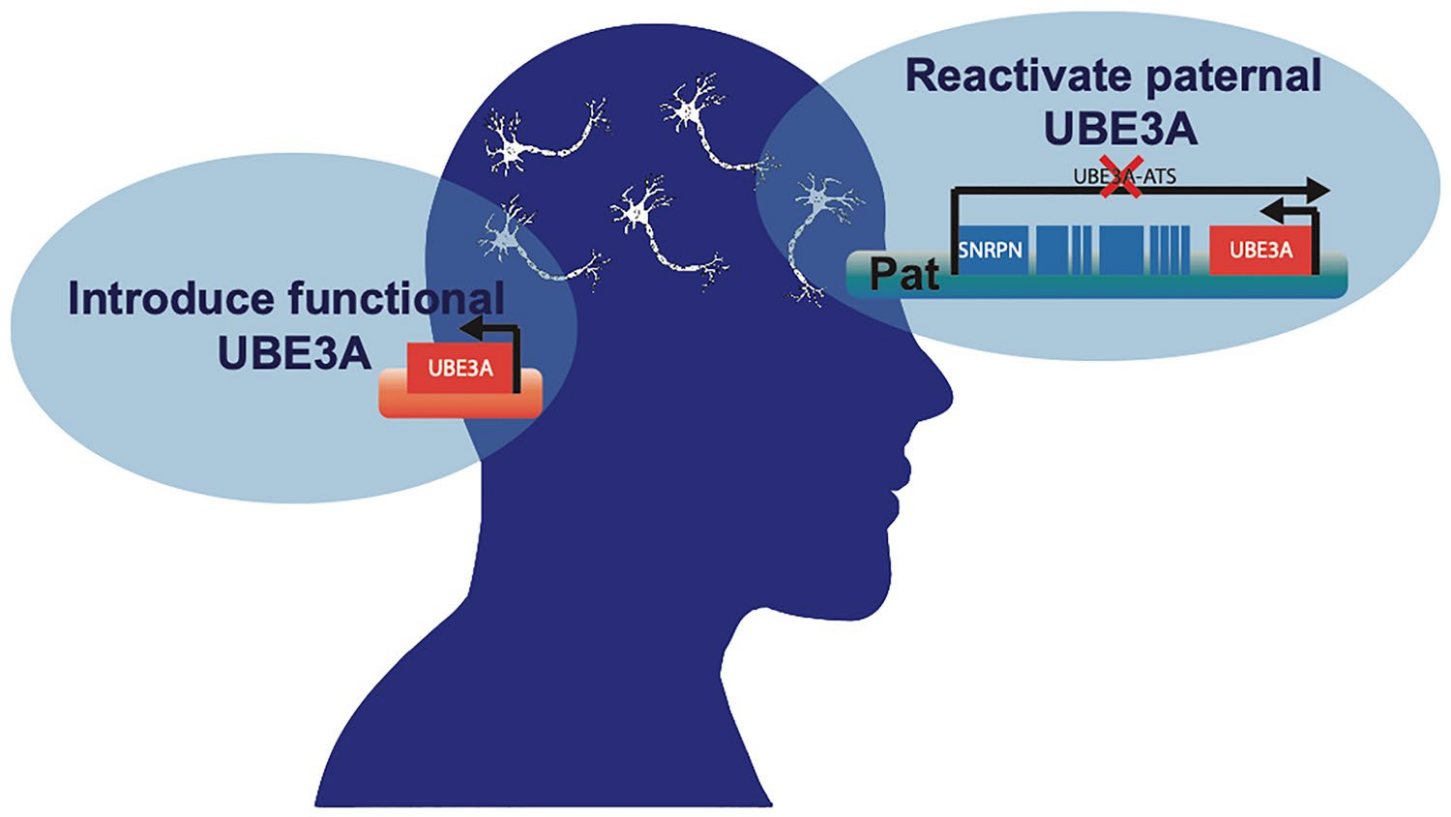
Gene therapy strategies for treating Angelman syndrome. The two most promising strategies to treat AS via gene therapy are to either introduce a functioning copy of UBE3A or reactivate the paternal allele by knockdown of the antisense transcript

In therapeutic development, the use of animal models is paramount in assessing both the safety and efficacy of a proposed therapeutic before advancement to regulatory processes and clinical trials. In vitro studies help to answer and investigate alterations at the cellular level and mechanistic questions [61–64], but in vivo work allows for the observation of therapeutic efficacy in a live, behaving animal. Behavioral domains relevant to AS include motor function, cognitive ability, sleep, developmental delay and seizures. Alleviation of seizures and their appearance behaviorally and via electroencphalographic (EEG) in the models is of particular importance, due to the high prevalence in the clinical AS population. Additionally, it is necessary to observe in model systems gross neuroanatomical abnormalities, fine grained histopathology, and electrophysiological patterns as they would be difficult or impossible to investigate in human patients. Finally, studies aimed to determine toxicity, safety, and dosage of therapeutics must also be conducted in vivo to observe how the therapy affects the whole organism. There are several animal models currently being used in AS research with various strengths and weaknesses that reflect their ability to model the disorder and the possibility of serving as tools to advance a therapy from bench to bedside (Fig. 2).

### Mouse Models of AS

Several *Mus musculus* lines have been generated to model AS by targeting mouse chromosome 7, the homolog to human chromosome 15’s location of *UBE3A,* using technically innovative designs including duplications of the paternal chromosome, ligase focused mutations, and large chromosomal deletions [65, 66]. As the majority of AS patients have a large deletion of 15q, an appropriate in vivo tool for investigation includes a mouse model with a large chromosomal deletion from *Ube3a* to *Gabrb3* [67]. The most widely utilized mouse model of AS has a deletion in exon 2 resulting in a loss-of-function mutation of *Ube3a* [25, 68–73]. This deletion results in offspring that could inherit the deletion from either the dam (*Ube3a*^*m−/p*+^) or sire (*Ube3a*^*m*+*/p−*^). Translational phenotypes including motor impairments detected by reduced latencies to fall off a beam and accelerating rotarod, while gait analysis demonstrated variability similar to kids with AS [74–80]. Interestingly, even with the robust motor deficit, normal sociability in a three-chambered social approach task was reported in *Ube3a*^*m−/p*+^ mice [70]. Learning and memory deficits were observed in ex vivo hippocampal slice long-term potentiation (LTP). LTP deficits specifically in the hippocampus indicated disruption of hippocampal calcium-calmodulin-dependent protein kinase II activity, which may result in decreased plasticity and learning and memory [81]. When this model was on the 129 background strain, it exhibited audiogenic-induced seizure susceptibility, that was background strain dependent [82–84], and abnormal EEG characteristics, including elevated delta frequency power, spiking activity and slow wave discharges, comparable to those seen clinically [85–87]. Moreover, sleep deficits such as reduced total sleep time, longer latencies to sleep onset and abnormal spindle production seen in the clinical population are consistently recapitulated in *Ube3a*^*m−/p*+^ mice, across strains [77]. Importantly, the exon-2 deletion model is *Ube3a* specific, on a C57BL/6 J background strain and [88, 89] exhibits intact imprinting throughout the brain [90].

Another unique model is a reporter mouse that does not delete or mutate *Ube3a,* rather it inserts a visible tag via fusion of yellow fluorescent protein (YFP) to the c-terminus of E6-AP [91]. These YFP reporter mice allow for the visualization of *Ube3a* from either the maternal or paternal copy. Reporter mice have become invaluable for translational neuroscience [92, 93].

### Rat Model of AS

Recently, a rat model of AS was created using a CRISPR/Cas9 system to delete the entire 90-kb *Ube3a* gene region [76, 78]. Impairments in neonatal ultrasonic vocalizations and reflexes were detected. Adult rats exhibited motor and sensory deficits by reduced vertical activity in an open field assay, faster latencies to fall off of an accelerating rotarod, and longer times for adhesive removal. Learning and memory was diminished in the *Ube3a*^*m−/p*+^ animals compared to wildtype littermate controls on a pairwise discrimination touchscreen task. Lastly, deletion animals demonstrated reduced exploration to affiliative pro-social calls, suggesting lower social communication or social cognition, currently being explored further [76]. Genetically engineered rat models are becoming a widely feasible investigative approach for preclinical research, as they provide enhanced behavioral capabilities relative to mice and more analogous pharmacological properties to humans [94–98].

## Small Molecule Drugs in the Treatment of Angelman Syndrome

Multiple small molecules are in clinical or pre-clinical development for Angelman syndrome. While precision therapy approaches may offer high future potential for disease-modifying treatment of genetically defined disorders, our understanding of these valuable therapies is relatively new. Indeed, at the time of writing, only two gene therapy products are currently FDA approved (Zolgensma® for spinal muscular atrophy with bi-allelic mutations in the SMN1 gene, and Luxturna® for bi-allelic RPE65 mutation-associated retinal dystrophy). Small molecule therapies, while not being disease modifying, can have great impact on specific symptom domains, such as seizures, having a major impact on quality of life. There is a large volume of preclinical and clinical knowledge around small molecule anticonvulsant drugs which can be leveraged for seizure control in AS.

### OV-101

OV-101 is a small molecule extrasynaptic GABA_A_ receptor agonist. It is also known as THIP (4,5,6,7-tetrahydroisoxazolo[5,4-c]pyridin-3-ol) or gaboxadol. As compared to other GABAergic drugs such as benzodiazepines, which act on synaptic GABA_A_ receptors mediating fast phasic inhibition, OV-101 acts specifically on extra-synaptic GABA_A_ receptors [99]. These receptors are activated by ambient GABA levels and produce tonic inhibition—a consistent low amplitude hyperpolarizing current, which results in overall decreased excitability of neurons (for review, see [100]. There is evidence in AS animal models that tonic inhibition is impaired [101]. There is evidence that the underlying mechanism is decreased ubiquitin-mediated degradation of GAT1, leading to an excess of GAT1 mediated uptake of GABA [102], which reduces ambient GABA spillover required to activate extra-synaptic GABA receptors mediating tonic inhibition. This has been linked to phenotypes such as ataxia [102] and EEG abnormalities [103]. OV-101 aims to correct that deficit by increasing tonic inhibition in the brain.

Preclinically, OV-101 has been shown to improve tonic inhibitory deficits in slice preparations from AS mice [102, 103]. It was also seen to improve deficits in motor coordination, by the rotarod [102]. In clinical studies, promising phase 2 data showed improvements in a constellation of symptom domains including sleep, motor function, communication abilities, challenging behavior, and anxiety as assessed by the CGI-I [104]. Unfortunately, this failed to translate to any significant benefit over placebo in phase 3, where OV-101 showed 0.7 point improvement compared to 0.8 point improvement in the placebo group (Ovid Therapeutics press release). Development of OV-101 is currently on hold pending further analysis of the phase 3 data.

### Trofinetide NNZ-2566 and NNZ-2591

Both trofinetide (NNZ-2566) and the follow-on molecule NNZ-2591 are small molecule IGF-1 mimetics. They activate PI3K-AKTt-mTOR and Ras-MAPK-ERK pathways and have been shown to increase synapse number and synaptic plasticity [105, 106]. Spine numbers have been shown to be reduced in AS mouse models [91] and activity-dependent ERK phosphorylation and synaptic plasticity are impaired [107–110]. The therapeutic hypothesis is that through upregulating synaptic plasticity and synapse number, these compounds can have benefit in AS.

There are no preclinical studies published using AS models for these compounds. However, NNZ-2591 showed positive effects on memory in scopolamine challenged rats [111], and NNZ-2566 showed anticonvulsant effects in an ischemia model [112], suggesting that these compounds may have benefits on cognition and seizure control. IGF-1 and IGF-1 mimetics such as trofinetide are being pursued for a number of neurodevelopmental disorders [113]. IGF-1 was seen to improve defective AMPA receptor-mediated neurotransmission, LTP and improve motor performance in a Phelan–McDermid syndrome model [114]. IGF-2 has shown beneficial effects in AS mice, improving cognitive and motor deficits, and attenuating audiogenic seizures [115]. However, some caution should be taken in interpreting IGF-2 data in support of trofinetide and NNZ-2591, as this ligand shows some non-specificity in binding both the IGF-1 and IGF-2 receptors. Clinically, NNZ-2566/Trofinetide has shown some level of benefit in phase 2 studies in Rett syndrome [116] and fragile X syndrome [117]. At the time of writing, NNZ-2591 has just completed a successful phase 1 trial, and clinical trials in AS are planned to start in 2021.

### Lovastatin

Lovastatin was the first compound to be marketed in the statin class of compounds. These are HMG-CoA reductase inhibitors, with broad lipid-lowering effects, commonly prescribed for hypercholesterolemia. Lovastatin also acts to reduce RAS/ERK signaling [118, 119] and decrease protein synthesis via inhibition of cap-dependent translation [120]. There is general interest in the use of statins to treat a number of neurodevelopmental disorders (reviewed in detail in [121]). In AS, excessive levels of synaptic proteins have been seen [2], and so it can be hypothesized that decreasing protein synthesis by lovastatin could show clinical benefit. Lovastatin has been seen to have effects on seizure control and learning and attention in preclinical models of neurodevelopmental disorders [122, 123], so could potentially have effects on multiple symptom domains in AS. A large amount of literature was reviewed on statins in neurodevelopmental disorders [121].

In a preclinical study, lovastatin protected AS mice from audiogenic seizures, and reduced long burst firing in slices from these mice [124]. This suggests that there could be an anticonvulsant effect of lovastatin in AS. A related compound, simvastatin, also improved cognitive and social function in an AS mouse model. The authors related this effect to mechanistically restoring HDAC1/2 activity in these mice [125]. Clinically, lovastatin failed to improve outcome measures in neurofibromatosis type 1 [126] and fragile X [127] but no clinical study has yet been conducted in AS.

### Minocycline

Minocycline is a tetracycline antibiotic which can be used to treat both gram-positive and gram-negative bacteria. Tetracycline antibiotics work by inhibiting protein synthesis [128], so could have potential benefit in AS by correcting excessive levels of synaptic proteins that have been spared ubiquitin mediated degradation. Minocycline also has activity as a matrix-metalloproteinase 9 (MMP9) inhibitor [129]. Studies in fragile X mice showed that minocycline increases the number of mature “mushroom” spines via its action as an MMP9 inhibitor [130]. Minocycline could be hypothesized to have a beneficial effect on synaptic morphology and plasticity in AS.

Clinically, Grieco et al. [131] showed some benefit in a small open-label trial in AS patients. However, a larger randomized placebo-controlled study revealed no benefit [132]. Subsequent work in a thorough in vivo test battery using AS mouse models also revealed no benefit of minocycline in AS preclinically [133].

### LB-100/PP2A Inhibitors

LB-100 is a protein phosphatase 2A (PP2A) inhibitor. Recently, PP2A was shown to be overactive in Ube3a^m−/p+^ mice as a result of loss of ubiquitin-mediated degradation of PTPA, a PP2A activator [134]. The authors show that overexpression of this important regulator in neurons phenocopied spine deficits seen in Ube3a^m−/p+^ mice, and genetically reducing expression of PTPA corrected spine morphology deficits. Pharmacologically, the authors showed that directly inhibiting PP2A using tool small molecule LB-100 was able to reduce PP2A activity, rescue reduced mEPSC frequencies, and improve motor behavior on both the wire suspension and rotarod tasks. While LB-100 is a tool compound and not a therapeutic, PP2A inhibition as an approach is chemically tractable, and represents an interesting new pharmacological target for AS. However, given its ubiquitous tissue expression, and involvement in many cellular processes, it remains to be seen if this approach can be tailored to specifically benefit AS patients.

### BK Potassium Channel Inhibitors

Sun et al. used a UBE3A-KO engineered human embryonic stem cell (hESC) model to demonstrate a biphasic effect on excitability, with KO neurons initially showing decreased spike frequency compared to wildtype neurons at low levels of current injection, but showing increased spike frequency at higher current levels [135, 136]. They interpreted the data to suggest that the effect was mediated by increased BK channel currents compared to wildtype. The increase in BK channel current resulted from increased BKα subunit expression, due to decreased ubiquitin mediated degradation. Paxilline, a toxic alkaloid BK channel antagonist, was able to reduce the aberrantly increased BK channel current levels. The authors demonstrated that paxilline could also normalize network dynamics in a 3D organoid model. Paxilline also showed anticonvulsant effects in flurothyl and picrotoxin induced seizures in AS mice. While paxilline itself is toxic, BK channel inhibition could be further investigated as a potential treatment for seizures in AS. Consistent with this, paxilline has also shown to have anticonvulsant effects in picrotoxin and pentylenetetrazol (PTZ) induced seizures in wild-type mice [137]. This remains an exciting potential new therapeutic avenue for AS and is being evaluated preclinically as a component of the Foundation for Angelman Syndrome for Therapeutics (FAST) infrastructure award.

## Precision Therapies in the Treatment of Angelman Syndrome

Gene therapy is simply the transfer of a therapeutic gene to treat a disease or disorder. This therapy can be performed ex vivo, in tissue collected then cultured in a lab, or in vivo*,* where the therapy is administered directly into the patient’s body. Precision therapy offers hope for many single-gene diseases in which conventional treatment has failed. Because AS is caused by the loss of functional UBE3A, gene therapies designed to introduce a working *UBE3A* copy or targeting the antisense silencing the maternal copy designed to reactivate the paternal allele are the most promising (Table 1). In 2019, GeneTx/Ultragenyx initiated a phase 1/2 ASO clinical trial. Early data in five patients showed minimal toxicity at low doses and symptomatic improvement by caregiver impression (CGI) analysis (Berry-Kravis unpublished). Another ASO compound is also in phase I clinical trial (Roche NCT04428281). The ASO approach is extremely promising but has some potential limitations. Delivery is typically intrathecal, which for Angelman children would require full anesthesia. The anesthesia procedure carries its own risks, and would need to be repeated 3–4 times each year for the life of the individual.

**Table 1:**
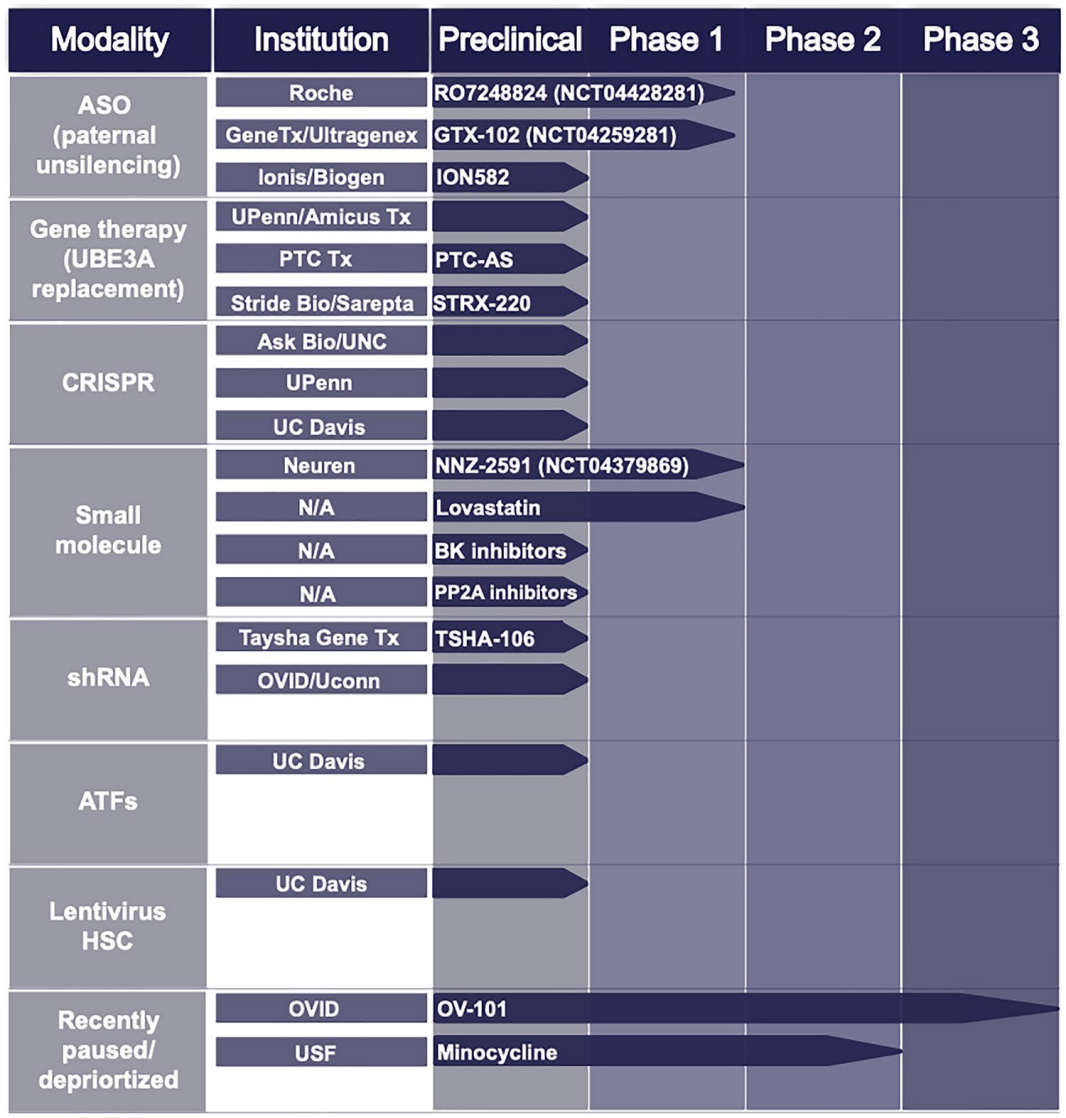
The current stage of various therapeutics in development for Angelman syndrome

### Therapies Targeted to Reactivate the Paternal UBE3A Allele by Suppressing UBE3A-ATS

Several studies have indicated that paternal *UBE3A* is silenced in neurons due to *UBE3A-*ATS [14, 20], and that suppression of the antisense transcript in cell and animal models results in the rescue of the phenotypic deficits reported [55, 138]. In one of the first attempts to silence the *Ube3a*-ATS, topotecan, a topoisomerase I inhibitor, was administered to cortical neurons collected from embryonic day 15.5 neonates with paternally inherited *Ube3a*-YFP [139]. Topotecan reactivated the paternal allele (as seen by an increase in fluorescence of YFP) by inhibiting transcriptional progression of the ATS. Additionally, results indicated that the ligase function of Ube3a was restored indicating functional rescue. When injected unilaterally into the ventricle of mice with paternal *Ube3a*-YFP, paternal expression was observed in the treated hemisphere lasting up to 12 weeks. Although topotecan was approved by the FDA for various cancers including ovarian and lung cancer [140], its lack of specificity and general toxicity has limited the advancement of the drug to the clinic. While topotecan is not a viable treatment for AS, this research paved the road for future studies focusing on paternal reactivation and highlighted the importance of specificity to a target gene.

Building from the topotecan results, one study aimed to reactivate the paternal allele by suppression of the *Ube3a*-ATS using a site-specific antisense oligonucleotide (ASO) approach [58]. ASO efficacy is dependent on (i) how well it targets the RNA of interest and (ii) construct modifications made directly to the ASO itself to prevent rapid degradation, promote affinity, and reduce toxicity. In this design, a phosphorothioate modified chimeric 2’-O-methoxyethyl DNA ASO was designed complementary to a region of mouse *Ube3a*-ATS downstream of SNRPN. After nuclear hybridization of the ASO to the ATS RNA, the *Ube3a*-ATS is cleaved and subsequently degraded. When the ASO was administered via a unilateral intracerebroventricular injection into a paternally inherited *Ube3a*-YFP mouse, paternal expression was reinstated for 4 months. Additionally, exon 2 deletion *Ube3a*^*m−/p*+^ mice treated with the ASO exhibited rescue of contextual fear conditioning deficits and excessive weight gain, albeit weight gain is not a translational phenotype in AS. To date, this is the only reported ASO used in an AS model, but with the progression of ASOs across clinical trials [141], it is expected that this will be a prominent therapeutic focus in future studies.

Various other engineered DNA-binding proteins such as zinc finger-based artificial transcription factors (Zinc-ATF), transcription activator-like effector-based artificial transcription factors (TALEs), and clustered regularly interspaced short palindromic repeat (CRISPR/Cas9) systems have been considered or studied as a tool to reactivate the paternal allele in AS [31]. Both Zinc-ATFs and TALEs use amino acid side chains to recognize DNA base pairs. These compounds activate or suppress a gene of interest by regulating its transcription. TALEs are more diverse in the spectrum of sequences they can target, but Zinc-ATFs have been more comprehensively studied and are therefore preferred in gene therapy design. A Zinc-ATF protein has been constructed and tested in an animal model of AS, with promising results [56, 57]. The construct was designed to target a site slightly upstream of SNRPN, and both subcutaneous and intraperitoneal injections resulted in widespread *Ube3a* expression in *Ube3a*^*−/p*+^ mice [56, 57]. Molecular therapies such as these are promising, but not yet feasible as therapeutic options due to the need for continued re-administration and lack of an optimized delivery method. Recently, two publications regarding the use of CRISPR/Cas9 nuclease to disrupt the *Ube3a-ATS* were described [142, 143]. In both studies, guide RNAs were used to target nuclease activity to a site-specific region of the region between Snord115. CRISPR/Cas9 is advantageous in its specificity and ease of site targeting using guide RNAs. While the targeting loci differed between the groups with the paper by Schmid et. al. designed to cause a single INDEL between *Snord115* and *Ube3a* 3′ UTR and the paper by Wolter et. al. designed to target approximately 75 regions of the *Snord115* cluster, both approaches disrupt *Ube3a-ATS* without affecting *Snrpn* or *Snord116* expression [142, 143]. Both strategies utilized AAV gene therapy for delivery of the Cas9 nuclease and guide RNA constructs. The Schmid group performed neonatal delivery and observed robust transduction efficiency and *Ube3a* upregulation. In utero injections were performed in the Wolter study with similar transduction efficiency and increased levels of *Ube3a.* Both studies utilized a variety of behavioral, anatomical, and functional assessments including body weight, brain size, open field, marble burying, nest building, open field and latency to fall on the rotarod task. In both studies, functional rescue was observed on multiple behavioral domains albeit on different tasks. These two manuscripts both represent the first in vivo nuclease use of Cas9 in the brain of an AS mouse model. While more work is needed to understand the optimal age of intervention, “optimal” translational outcome measures, and further studies on the safety and toxicity of this approach, they both present promising avenues to disrupt *Ube3a-ATS* and drive expression of *Ube3a* from the paternal allele.

### Therapies Designed to Introduce a Functional Copy of UBE3A

Given that AS is a single-gene disorder, resulting from the loss of UBE3A activity in the brain, another practical gene therapy approach would be to introduce functioning *UBE3A* into affected cells. This approach has been attempted by injecting an adeno-associated virus (AAV) carrying *Ube3a* into the hippocampus of Ube3a-deficient mice [144]. The treatment resulted in localized reinstatement of Ube3a to wild-type levels and rescue of the LTP deficit previously reported. Behaviorally, contextual fear conditioning and Morris water-maze impairments were also restored to that of wildtype littermate controls, supplementing the LTP finding and suggesting this treatment was effective for learning and memory. Motor deficiencies, measured by latency to fall off an accelerating rotarod and exploratory behavior in a novel field, were not improved. Anxiety-like behavior, measured by elevated plus-maze, also showed no treatment effect. The lack of improvement on these measures may be due to the limited distribution of the AAV throughout the hippocampus.

In a more recent study, modified autologous hematopoietic stem and progenitor cells with a lentiviral vector expressing *UBE3A* were introduced into immunocompromised *Ube3a*^*m−/p*+^ mice to provide functional UBE3A via cross correction [75]. This treatment resulted in behavioral rescue of several motor assays including open field, beam walking, rotarod, gait outcomes, including stride width, stride frequency and stance. Additionally, cognitive improvements were observed in novel object recognition and the persistent electrophysiological characteristic of elevated delta power typically seen in AS individuals and *Ube3a*^*m−/p*+^ mice and rats [77, 85, 86, 145–147] were reduced to that of wild-type levels. Of particular importance, all behavioral rescue was observed in both neonatal and adult treated mice, providing promising work for future cross correction type work in single gene disorders.

## Challenges in Designing a Precision Medicine for Angelman Syndrome

While targeted gene therapies offer many advantages in the treatment of AS, there are still challenges to be discussed when utilizing these methods. The ideal gene therapy for AS would be specific to *UBE3A*, minimally invasive, non-toxic, restore UBE3A to normal endogenous levels, and be effective at various developmental timepoints. One of the largest roadblocks faced in therapeutic development is delivery.

Delivery of a gene therapy to the required site is a challenge encountered universally. Given that AS is a CNS-specific disorder, delivery must be able to cross the BBB or be administered intracranially or intrathecally. Intracranial injections are invasive and generally not customary for a non-lethal disorder; therefore, intrathecal or peripheral administration are at the center in gene therapy design. Attention must be paid to the delivery vehicle as well (viral vectors, stem cells, liposomes, etc.) to reduce possible oncogenic or immune activation effects such as those seen in AAV research [148].

Another challenge a gene therapy designed for AS is that of gene dose. Maternally derived duplications or triplications of 15q11.2-q13.3 are the most penetrant genomic rearrangements observed in individuals with ASD, accounting for up to ~3% of ASD cases, and indicated in/responsible for Dup15q syndrome [149–151]. Pronounced clinical features associated with Dup15q syndrome are intellectual disability, seizures, anxiety, global developmental delay, hypotonia, speech impairments, motor coordination, and minor dysmorphic features [152–154]. While it has been reported that imprinting does not reduce the amount of UBE3A in neurons [155], it will be important to ensure that paternal reactivation or UBE3A/*UBE3A* reintroduction does not overexpress the gene.

Finally, the time point of a treatment is critical in a developmental disorder such as AS. Ideally, the hope is to treat as early as possible, but a therapy for all ages is essential. Previous rodent work has shown that a loss of *Ube3a* in early embryonic development resulted in predictable AS behavioral phenotypes while either juvenile or adult loss of *Ube3a* had fewer, lessened behavioral deficits, suggesting the importance of *Ube3a* reinstatement earlier in life [156, 157]. These studies have led to the discussion of prenatal treatment for AS [158], but this can be an invasive and dangerous suggestion especially when the severity of symptoms vary by individual. It is unclear what symptoms may be alleviated by various treatments given at different developmental timepoints. In fact, in a recent publication, using hematopetic stem cells to introduce a Ube3a-expressing lentiviral vector illustrated behavioural rescue of motor, cognitive and neurophysiological AS phenotypes following adult and neonatal-treated animals [75]. This design known as “cross correction” has been proven safe in other disorders, and is currently in IND review with the FDA. Other work by a collaborating group found adult rescue of AS behavioral phenotypes following systemic treatment with an artificial transcription factor (ATF) [56, 57] and yet another laboratory was successful with viral delivery of Ube3a [144].

## Conclusion

Here, we review the Angelman Syndrome, the causal gene, *UBE3A,* animal models of AS, emerging genetic, stem cell and precision medicine therapies and associated challenges faced in bench to bedside pipeline. Currently, there is no cure for AS and the treatments available are only useful in mitigating and managing a few of the symptoms throughout a patient’s lifetime. Gene and molecular precision therapies offer a promising glimpse into the future of precision-medicine and work in this field will likely pave the road toward therapeutic development for other single-gene disorders.

## Supplementary Information

Below is the link to the electronic supplementary material.

Supplementary file1 (PDF 372 KB)

Supplementary file2 (PDF 499 KB)

Supplementary file3 (PDF 508 KB)

Supplementary file4 (PDF 508 KB)
